# New insights into the comorbid conditions of Turner syndrome: results from a long-term monocentric cohort study

**DOI:** 10.1007/s40618-022-01853-z

**Published:** 2022-07-30

**Authors:** A. Gambineri, E. Scarano, P. Rucci, A. Perri, F. Tamburrino, P. Altieri, F. Corzani, C. Cecchetti, P. Dionese, E. Belardinelli, D. Ibarra-Gasparini, S. Menabò, V. Vicennati, A. Repaci, G. di Dalmazi, C. Pelusi, G. Zavatta, A. Virdi, I. Neri, F. Fanelli, L. Mazzanti, U. Pagotto

**Affiliations:** 1 Unit of Endocrinology and Diabetes Prevention and Care, IRCCS Azienda Ospedaliero-Universitaria Di Bologna, Via Massarenti 9, 40138 Bologna, Italy; 2 Pediatric Endocrinology and Rare Disease Unit, IRCCS Azienda Ospedaliero-Universitaria Di Bologna, Bologna, Italy; 3 Department of Biomedical and Neuromotor Sciences, University of Bologna, Bologna, Italy; 4 Genetic Unit, IRCCS Azienda Ospedaliero-Universitaria Di Bologna, Bologna, Italy; 5 Division of Dermatology, IRCCS Azienda Ospedaliero-Universitaria Di Bologna, Bologna, Italy

**Keywords:** Turner syndrome, Cardiovascular events, Type 2 diabetes, Cancer, Osteoporosis

## Abstract

**Purpose:**

Many questions concerning Turner syndrome (TS) remain unresolved, such as the long-term complications and, therefore, the optimal care setting for adults. The primary aim of this long-term cohort study was to estimate the incidence of comorbid conditions along the life course.

**Methods:**

A total of 160 Italian patients with TS diagnosed from 1967 to 2010 were regularly and structurally monitored from the diagnosis to December 2019 at the University Hospital of Bologna using a structured multidisciplinary monitoring protocol.

**Results:**

The study cohort was followed up for a median of 27 years (IQR 12–42). Autoimmune diseases were the comorbid condition with the highest incidence (61.2%), followed by osteoporosis and hypertension (23.8%), type 2 diabetes (16.2%) and tumours (15.1%). Median age of onset ranged from 22 years for autoimmune diseases to 39 years for type 2 diabetes. Malignant tumours were the most prominent type of neoplasm, with a cumulative incidence of 11.9%. Papillary thyroid carcinoma was the most common form of cancer, followed by skin cancer and cancer of the central nervous system. Only one major cardiovascular event (acute aortic dissection) was observed during follow-up. No cases of ischaemic heart disease, heart failure, stroke or death were recorded.

**Conclusions:**

This cohort study confirms the need for continuous, structured and multidisciplinary lifelong monitoring of TS, thus ensuring the early diagnosis of important comorbid conditions, including cancer, and their appropriate and timely treatment. In addition, these data highlight the need for the increased surveillance of specific types of cancer in TS, including thyroid carcinoma.

**Supplementary Information:**

The online version contains supplementary material available at 10.1007/s40618-022-01853-z.

## Introduction

Research on Turner syndrome (TS) continues to reveal new facts and insights [1]; however, many questions remain unresolved, such as the long-term complications and, therefore, the optimal care setting for adults. Further questions involve the long-term impact of non-modifiable factors, such as the karyotype together with modifiable factors, such as hormone replacement therapies with growth-hormone (GH) or with oestrogen–progestins (EPs), both frequently prescribed to patients with TS [1, 2]. GH treatment is often administered to patients with TS during infancy to increase height, and EP therapy is usually prescribed to initiate and sustain sexual maturation and to reduce the risks posed by hypogonadism [2].

Several studies have reported an increased morbidity in TS patients due to autoimmune disorders, particularly thyroiditis, diabetes, osteoporosis, hypertension, cardiovascular (CV) diseases and diseases of the digestive system [1, 3–5]. In addition, a loss of lifespan of 13–15 years has been reported in TS compared with the general population [6] with most of the excess mortality attributed to CV diseases [7–9].

However, these findings mostly derive from cross-sectional or retrospective studies or from studies based on registries. The few prospective studies available have a small sample size and a short follow-up period. This suggests a potential bias in the estimation of the long-term comorbidities, particularly in adults.

Another still unresolved question due to the few data available is whether the overall risk of cancer in TS differs from that of the general population. However, there is agreement regarding the different pattern of tumour occurrence in TS, with an increased risk of benign central nervous system (CNS) tumours, particularly meningioma and benign skin neoplasms and skin cancer, particularly melanoma, a decreased risk of breast cancer, and an increased risk of gonadal tumours in patients with Y chromosome sequences [10–14].

Very few data are available on thyroid carcinoma, and no specific screening monitoring protocol is advised in the current official guidelines [1, 15].

This paper presents a long-term cohort study in which a large population of patients with TS was regularly and structurally monitored from diagnosis to late adulthood in the same hospital using a multidisciplinary monitoring protocol.

The primary aim was to estimate the incidence of hypertension, type 2 diabetes, autoimmune diseases, tumour, osteoporosis, and major CV events. The secondary aim was to investigate the relationship of karyotype, menstrual pattern (spontaneous cycles, primary or secondary amenorrhea), GH and EPs therapy with comorbid conditions. We believe that this study provides key information to guide clinicians on the appropriate long-term monitoring of TS.

## Materials and methods

### Study design and patients

In this prospective cohort study, 160 Italian patients were consecutively enrolled soon after the diagnosis of TS was performed by chromosome karyotyping at the University Hospital of Bologna, Italy from 1967 to 2010. All patients in the study were regularly monitored at the S. Orsola University Hospital by a team of paediatricians from the Paediatric Clinic (ES, AP, FT, LM) during the paediatric age up to the transition age, and then by a team of endocrinologists from the Endocrinology Unit (AG, PA, FC, CC, PD, EB, DIG) from the transition age to the end of the follow-up (December 2019). The study protocol for monitoring TS was followed by the Paediatric Clinic and the Endocrinology Unit.

After the diagnosis of TS, a yearly follow-up was conducted, with a comprehensive medical and family history and a physical examination including anthropometry and blood pressure (BP) measurement. Height and body weight were measured using standardized procedures, and the BMI was calculated [16]. BP was measured twice in the morning during the clinical visit and, when high BP levels were recorded, home BP monitoring was required. A fasting blood sample was also taken once a year in the morning, between 8:00 and 9:00 a.m.

Laboratory tests included blood count, total cholesterol, high-density lipoprotein cholesterol, triglycerides, glucose, glycated haemoglobin (HbA1c), creatinine, liver function tests, thyroid hormones, serum electrolytes, proteinogram, and urine analysis. Serum thyroid antibodies and transglutaminase antibodies were performed every 2 years if the previous evaluation was negative. A 75 g oral glucose tolerance test (OGTT) was also performed every 2 years, starting at 18 years until a diagnosis of diabetes was made.

Patients with diabetes were tested for serum c-peptide once a year, and for anti-glutamic acid decarboxylase (GAD) and anti-islet cell antibodies every 2 years. Each patient underwent a dermatological examination once a year. Abdominal and pelvic ultrasound, breast ultrasound or mammogram, cardiology visit with resting electrocardiogram (ECG) plus transthoracic echocardiography (TTE), and audiometric evaluation were performed every 3 years. Lumbar spine and femoral dual energy X-ray absorptiometry (DXA) scan, and magnetic resonance scan (CMR) of the thoracic aorta were performed every 5 years (DXA monitoring was started at the age of 18 years). Finally, a thyroid ultrasonography was performed every 2 years, starting at 18 years.

All these tests were performed earlier if needed, and specific diagnostic investigations were carried out in the case of a suspected pathology. Hypertension and diabetes were diagnosed according to current guidelines at the time of each assessment in adults and children [17–25]. Osteoporosis was defined by a bone mineral density (BMD) T score≤-2.5 in at least one of the two regions analysed (lumbar spine or femoral neck/total hip) or by a fragility fracture (i.e. due to low-energy trauma), which was clinically suspected and radiologically confirmed [26]. Diagnosis of Hashimoto’s thyroiditis (HT) was made if antibodies anti-thyroperoxidase (TPO) and/or anti-thyroglobulin were detected in serum [27], whereas a diagnosis of Graves' disease was made in the presence of hyperthyroidism with TSH-receptor antibodies (TRAb) in the circulation [28]. Type 1 diabetes was diagnosed in the presence of low c-peptide and pancreatic β-cell antibodies in the circulation [29]. Coeliac disease, inflammatory bowel disease and chronic atrophic gastritis were confirmed by biopsy, and tumour by histology. Ischaemic heart disease and stroke, heart failure and aortic dissection were considered as major CV events.

A team of specialists (including a dermatologist, cardiologist, radiologist, otolaryngologist, gynaecologist) was involved in the multidisciplinary monitoring of the patients throughout the study. The study was approved by the Ethics Committee of the University Hospital of Bologna, and each participant gave their informed consent.

### Statistical analysis

The cumulative incidence and the incidence rate of comorbid conditions were estimated for the overall study population. For the secondary aim, the associations between categorical variables were analysed using the χ^2^ test. The prevalence by age groups at follow-up of hypertension, type 2 diabetes and osteoporosis was also estimated. In addition, the prevalence of each comorbid condition was compared among age groups and with national data for the year 2019 (https://www.istat.it/it/dati-analisi-e-prodotti/banche-dati/statbase) using the χ^2^ test (Supplemental Table 1).

The significance level was set at p < 0.05. Statistical analyses were conducted using IBM SPSS, v. 25.0.

## Results

The study cohort consisted of 160 TS women, 90.6% of whom were recruited during childhood (mean age = 9.4 years, SD = 7.1) and followed up for a median of 27 years (IQR 12–42). The last follow-up was carried out in December 2019. The characteristics of the cohort are provided in Table 1.

**Table 1:** Characteristics of the study cohort of 160 women with Turner syndrome

Variable	N (%) or mean (SD)
Age at follow-up (mean and SD, years)	37.1 (8.8)
Age at follow-up (age range, years)	
21–34	66 (41.3%)
35–44	60 (37.5%)
45–66	34 (21.3%)
Age at diagnosis (age range, years)	
< 18	145 (90.6%)
18–24	9 (5.6%)
^ 3^25	6 (3.8%)
Karyotype	
45,X	58 (36.3%)
Mosaic 45,X/46,XX	20 (12.5%)
Isochromosome (Xq)	30 (18.8%)
46,Xi(Xq)	
45,X/46,Xi(Xq) mosaic	
Mosaic 45,X/46,XY	17(10.6%)
Others	35 (21.9%)
Ring X	
Complex	
Partial X Deletions	
Menstrual pattern	
Spontaneous cycles	20 (12.5%)
Secondary amenorrhea	13 (8.1%)
Primary amenorrhea	127 (79.4%)
GH therapy	
No	47 (29.4%)
Yes	113 (70.6%)
EPs therapy	
No	12 (7.5%)
Yes HRT	77 (48.1%)
Yes OCT	71 (44.4%)

*GH* growth hormone, *EPs* oestrogen–progestins, *HRT* hormonal replacement therapy, *OCT* oral contraceptive therapy

A total of 113 patients (70.6%) received recombinant human GH therapy on average for 6.7 ± 3.1 years at a dose of 0.33 mg/kg per week, for seven days. GH therapy was started as soon as growth failure was demonstrated and continued until little growth potential remained (growth velocity < 2 cm/year). Mean age at starting therapy was 9.6 ± 3.1 years and the average age at discontinuation was 16.4 ± 1.6 years.

A total of 148 patients were treated with EPs. The remaining 12 patients did not receive EPs therapy, either because of spontaneous menstrual cycles (9 patients) or because of adverse side effects soon after the beginning of treatment (3 patients). Of the women treated with EPs, 77 used oral or transdermal estradiol together with a synthetic progestin at doses compatible with replacement therapy (hormonal replacement therapy; HRT), whereas 71 used ethinylestradiol together with synthetic progestin (oral contraceptive therapy; OCT). EPs therapy was started at a mean age of 16.2 ± 2.4 years in the HRT group and 16.5 ± 2.4 years in the OCT group. Only four out of the 148 patients on EPs discontinued the therapy during follow-up because they reached menopausal age (50–52 years).

**Fig. 1:**
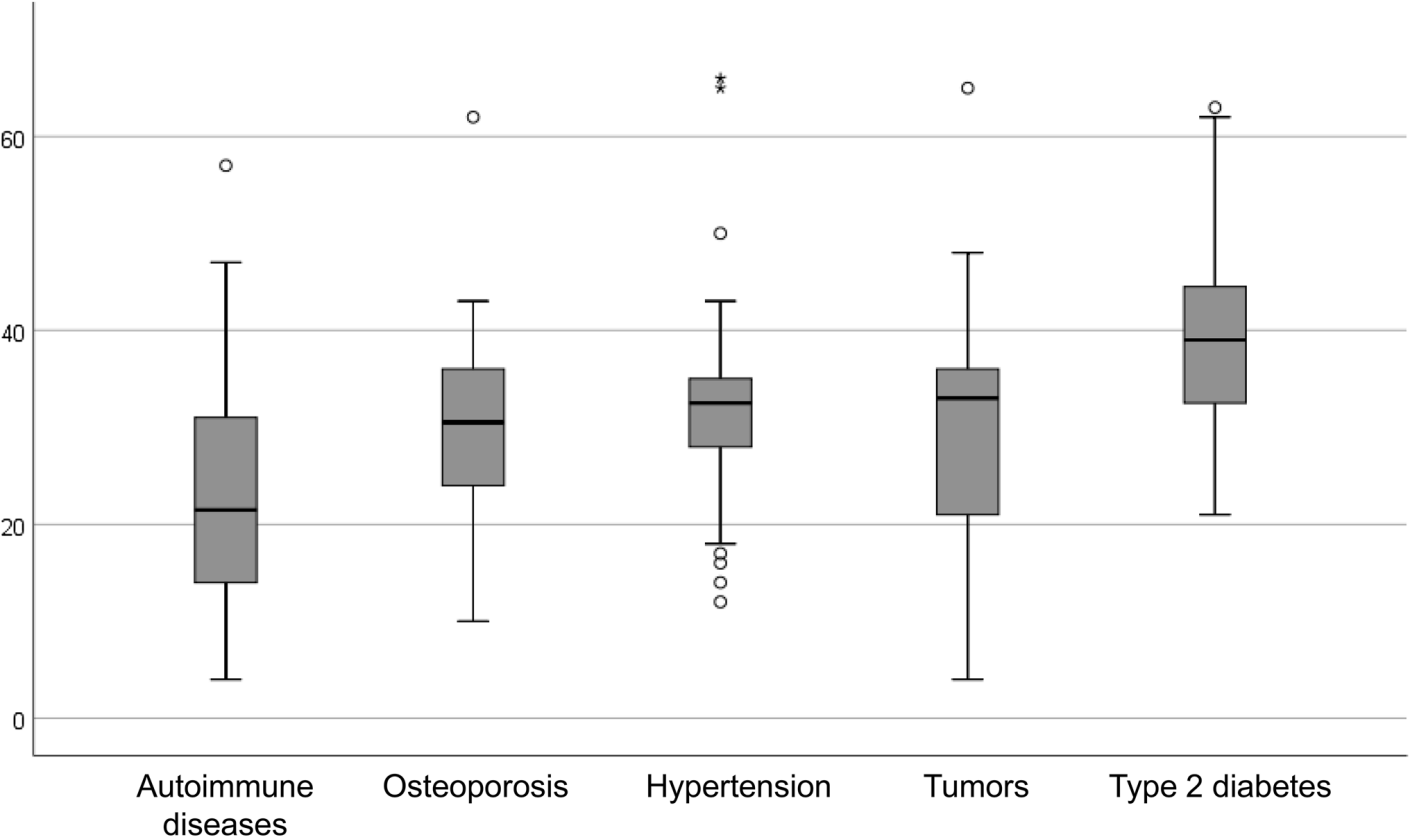
Boxplots showing the age of onset of comorbid conditions

The cumulative incidence and incidence rate of comorbid conditions are shown in Table 2. The onset age of comorbid conditions is shown in Table 2 and Fig. 1. Autoimmune diseases were the comorbid conditions with the highest incidence (61.2%), followed by osteoporosis and hypertension (23.8%), then by type 2 diabetes (16.2%) and tumours (15.1%). HT was the most common autoimmune comorbidity. Overall, 89 patients developed HT, 79 in isolated form and 10 combined with other autoimmune diseases (4 cases with coeliac disease, 3 cases with alopecia areata, one case with Graves’ disease, one case with type 1 diabetes, and one case with Sjogren’s syndrome). The other 9 cases of autoimmune diseases included coeliac disease, psoriasis, inflammatory bowel disease, chronic atrophic gastritis, Sjogren’s syndrome, Graves’ disease and vitiligo, in isolated or combined forms. During follow-up, 20 patients developed tumours, and 3 patients developed multiple tumours (skin melanoma plus paraganglioma, papillary thyroid carcinoma plus breast carcinoma, papillary thyroid carcinoma plus oligoastrocytoma plus diffuse astrocytoma). Malignant tumours (including thyroid carcinoma, renal cell cancer, skin cancer, breast cancer, ovarian cancer and CNS tumours) were the most prominent form, with a cumulative incidence of 11.9% and an incidence rate of 0.44 per 100 person-year. Thyroid carcinoma (histologically all papillary in our cohort) was the most common form of cancer with a cumulative incidence of 5% and an incidence rate of 0.18 per 100 person-year. Table 3 shows the type of tumour and age of diagnosis. Table 4 details some characteristics of the papillary thyroid carcinomas.

**Table 2:** Incidence of comorbid conditions

Incident comorbidities	Cumulative incidence	Incidence rate (100 persons/year)	
Autoimmune diseases	98 (61.2%)	3.31	
Age of onset (mean ± SD)	23 ± 10.2		
Osteoporosis	38 (23.8%)	1.23	
Age of onset (mean ± SD)	29.9 ± 9.9		
Hypertension	38 (23.8%)	0.94	
Age of onset (mean ± SD)	32.4 ± 11.2		
Type 2 diabetes	26 (16.2%)	0.60	
Age of onset (mean ± SD)	39.9 ± 9.4		
Tumours			
One tumour	17 (10.7%)		
Multiple tumours	3 (1.9%)		
Any tumour	24 (15.1%)	0.56	
Age of onset of the first tumour (mean ± SD)	33.7 ± 12.4		
Malignant tumours	19 (11.9%)	0.44	
Benign tumours	5 (3.1%)	0.12	

1 patient with type 2 diabetes, 3 with autoimmune diseases and 1 with tumour (acute lymphoblastic leukaemia) prior to the diagnosis of Turner's syndrome were excluded from the incidence count

**Table 3:** Type of tumour and age of diagnosis

Type of tumour (N of cases)	Age of diagnosis (years)
Malignant tumours (19)	
Thyroid carcinoma, papillary (8)	36;33;21;36;32;21;25;27
Renal cell cancer (1)	35
Skin cancer	
Malignant melanoma (2)	48;46
Basal cell carcinoma (2)	65;43
Breast cancer (1)	46
Ovarian cancer*	
Dysgerminoma (1)	16
Malignant CNS tumours (4)#	26;26;34
Benign tumours (5)	
Benign CNS tumours (4)§	43;35;21;52;31
Benign ovarian tumour*	
Gonadoblastoma (1)	12

^#^ 1 case of oligoastrocytoma, 1 case of diffuse astrocytoma, 1 case of aggressive vertebral hemangioma and 1 case of malignant schwannoma

^§^ 2 cases of neurinoma, 1 case of paraganglioma and 1 case of meningioma

^*^Both with Y chromosome material

**Table 4:** Papillary thyroid carcinomas: ultrasonic features, cytologic diagnosis, age at diagnosis, staging, and treatment

Case	Ultrasonic features	Cytologic diagnosis	Age at diagnosis (yrs)	Staging (AJCC 8th Edition)	Treatment	Other cancers	GH therapy	HT
#1	Single hypoechoic nodule of 10 mm with microcalcification in the right lobe and metastatic lymph nodes in the omolateral neck	TIR4	21	pT1bN1aMx	Total thyroidectomy + radioiodine	No	Yes	No
#2	Single hypoechoic nodule of 12 mm with microcalcification in the right lobe	TIR5	33	pT1aN0bM0 mETE	Total thyroidectomy + radioiodine	Yes; Breast cancer at 46 yrs	Yes	Yes
#3	Single hypoechoic nodule of 13 mm with microcalcification in the left lobe	TIR5	36	pT1aN0aMx mETE	Total thyroidectomy + radioiodine	No	Yes	No
#4	Single hypoechoic nodule of 10 mm with microcalcification in the left lobe	TIR3B	32	pT1aN0bMx mETE	Emithyroidectyomy	No	No	No
#5	Bilateral nodules; the biggest of 10 mm in the right lobe	TIR4	25	pT1bN1aMx mETE	Total thyroidectomy + radioiodine	No	Yes	Yes
#6	Single hypoechoic nodule of 6 mm with microcalcification in the left lobe in familiarity for thyroid carcinoma	TIR4	36	pT1aN0aM0 mETE	Emithyroidectyomy	No	No	No
#7	Single hypoechoic nodule of 10 mm with microcalcification in the left lobe	TIR4	21	pT1aN0bMx	Emithyroidectyomy	Yes; Oligoastrocytoma at 26yrs	Yes	Yes
#8	Bilateral nodules; the biggest of 12 mm in the right lobe	TIR3B	27	pT1a(m)N0bMx	Emithyroidectyomy first, followed by total thyroidectomy + radioiodine	No	Yes	Yes

*AJCC* American Joint Committee on Cancer, *mETE* minimal extrathyroidal extension, *HT* Hashimoto’s thyroiditis

The median age of onset of comorbidities ranged from 22 for autoimmune diseases, to 39 for type 2 diabetes.

Overall, 131 patients (81.9%) had at least one comorbidity. The most common comorbidity profiles were autoimmune diseases alone (46, 28.7%), autoimmune diseases with osteoporosis (13, 8.1%), autoimmune diseases with hypertension (11, 6.9%), osteoporosis only (8, 5%), autoimmune diseases with tumour (7, 4.4%).

Seven out of the 38 patients with osteoporosis developed fragility fractures, all in the trabecular bones.

When we analysed the relationship between karyotype, menstrual pattern, GH and EPs therapy with comorbid conditions, we found that patients with karyotype 45,X were more likely to have hypertension, while those with mosaic 45,X/46,XX or 45,X/46,XY were less likely to have it (*χ*^2^ = 13.6, *p* = 0.009) (Table 5). In addition, patients with spontaneous menstrual cycles were less likely to have osteoporosis (*χ*^2^ = 7.2, *p* < 0.05) and patients treated with GH were less likely to have type 2 diabetes (*χ*^2^ = 7.9, *p* = 0.005) (Table 6).

**Table 5:** Prevalence of comorbid conditions by karyotype; *p* values for chi-square test

		Hypertension	Type 2 diabetes	Autoimmune diseases	Tumours	Osteoporosis
Karyotype	45,X	21	12	36	6	11
	Mosaic 45,X/46,XX	55.3%	44.4%	35.6%	28.6%	28.9%
		2	1	13	1	4
		5.3%	3.7%	12.9%	4.8%	10.5%
	Isochromosome (Xq)	5	3	22	7	10
		13.2%	11.1%	21.8%	33.3%	26.3%
	Mosaic 45,X/46,XY	0	1	6	3	5
		0.0%	3.7%	5.9%	14.3%	13.2%
	Others	10	10	24	4	8
		26.3%	37.0%	23.8%	19.0%	21.1%
Total	38	27	101	21	38	
	*p* = 0.009	*p* = 0.075	*p* = 0.112	*p* = 0.321	*p* = 0.605

**Table 6:** Prevalence of comorbid conditions by type of menstrual cycle, EPs and GH treatment; *p *values for chi-square test

	Hypertension		Type 2 diabetes		Autoimmune diseases		Tumours		Osteoporosis	
	*N*	%	*N*	%	*N*	%	*N*	%	*N*	%
Menstrual pattern										
Spontaneous cycles	3	15.0%	1	5.0%	15	75.0%	1	5.0%	0	0.0%
Secondary amenorrhea	3	23.1%	3	23.1%	7	53.8%	1	7.7%	4	30.8%
Primary amenorrhea	32	25.2%	23	28.1%	79	62.2%	19	15.0%	34	26.8%
		*p* = 0.608		*p* = 0.286		*p* = 0.419		*p* = 0.393		*p* = 0.027
EPs										
No	2	16.7%	2	16.7%	9	75.0%	1	8.3%	1	8.3%
Yes HRT	22	28.6%	13	16.9%	49	63.6%	12	15.6%	13	16.9%
Yes OCT	14	19.7%	12	16.9%	43	60.6%	8	11.3%	24	33.8%
		*p* = 0.376		*p* = 1		*p* = 0.626		*p* = 0.649		*p* = 0.023^§^
GH										
No	14	29.8%	14	29.8%	28	59.6%	8	17.0%	13	27.7%
Yes	24	21.2%	13	11.5%	73	64.6%	13	11.5%	25	22.1%
		*p* = 0.247		*p* = 0.005		*p* = 0.548		*p* = 0.347		*p* = 0.454

^§^ In a multiple logistic regression model using Firth’s procedure, EPs was unrelated to osteoporosis after adjusting for menstrual cycles

Major CV events included only one case of acute aortic dissection, while no cases of ischaemic heart disease, stroke or heart failure were recorded. None of the patients died.

## Discussion

This long-term monocentric cohort study involving a large number of TS patients followed regularly from diagnosis for a median of 27 years enabled us to estimate the incidence of various complications of TS over the life course, the genotype–phenotype association, and the long-term impact of GH and EPs therapy.

This study confirms that TS is complicated by autoimmune diseases, hypertension, type 2 diabetes, osteoporosis and also tumours, particularly cancer and mainly papillary thyroid carcinoma, and that, with the exception of autoimmune diseases, these complications mainly appear in adulthood.

As major CV events, we found only one case of acute aortic dissection which was promptly and successfully treated with surgery and no cases of ischaemic heart disease, stroke or heart failure. This is in contrast with other studies evaluating long-term complications in TS, which reported major CV events [8, 9] as the main complications during adulthood and the main cause of death of this population. A possible explanation for this discrepancy is that our study population was on average younger at follow-up than the populations of the other two studies. However, the literature data show that lean and normotensive women with TS whose condition is also well controlled with hormone replacement therapy might never develop ischaemic heart disease, whereas hypertensive patients with TS and with insufficient hormone replacement therapy, obesity and type 2 diabetes are at very high risk of developing stroke or myocardial infarction [30, 31].

As in the general population, hypertension is an important risk factor for the development of aortic dissection and myocardial infarction or stroke in TS [1, 31, 32]. The strict multidisciplinary monitoring of our population and the appropriate and timely treatment of hypertension and the other CV risk factors, such as diabetes, timely prophylactic surgical intervention for ascending aortic dilation (2.5% of cases in our population) or for coarctation of the aorta (3.7% of cases in our population), and, probably, the long-term and appropriate EPs therapy prevented the development of major CV events. In fact, despite the high incidence in our population of hypertension (23.8%) and of type 2 diabetes (16.2%), as well as the high prevalence of congenital heart defects predisposing patients to aortic dissection (bicuspid aortic valve 8.1%) [31, 33, 34], there was only one case of aortic dissection and no cases of myocardial infarction, stroke, or heart failure.

The most common TS comorbidity in our study was autoimmune disease, and the most frequent form, in accordance with the literature, was HT [35–37]. In line with the literature, we found no cases of Addison’s disease nor type 1 or type 2 autoimmune polyglandular syndrome [38].

Various hypotheses have been put forward to explain the increased risk of autoimmunity in TS, but the exact aetiology remains uncertain [1]. In our study, no association of autoimmune diseases with karyotype, GH or EPs therapy, or menstrual patterns was found. The lack of a relationship between autoimmune diseases and EPs therapy or the menstrual pattern suggests that neither estrogens nor progesterone influence the development of autoimmune diseases in TS. This is in contrast with the general population, where a strong impact of sex hormones on autoimmunity has been proved [39].

In our study, EPs therapy had no impact on the development of osteoporosis, which was, however, influenced by the menstrual pattern. TS with spontaneous menstruation presented a normal BMD, whereas a high and similar prevalence of osteoporosis was found in TS with primary and secondary amenorrhea despite EPs treatment. In line with other studies, these findings suggests that in TS, the main factor related to osteoporosis is inadequate precocious oestrogen and androgen exposure which can only be guaranteed by an adequate ovarian function [40, 41]. However, it is important to underline that in our cohort, EPs therapy was initiated at a median age of 16 years. It is therefore possible that the relative late initiation of EPs therapy may have not positively influenced BMD sufficiently [42–44].

According to the literature, karyotype and GH therapy were not related to osteoporosis [40, 41, 45]. Conversely, patients treated with GH were less likely to develop type 2 diabetes in adulthood. Considering that patients with TS are at increased risk of type 2 diabetes and have a specific defect in glucose-stimulated insulin secretion [46], this is an extremely positive result. It highlights the potential long-term impact of GH therapy on the carbohydrate metabolism and supports the notion that during GH therapy patients' insulin sensitivity and carbohydrate tolerance are reduced. However, at its cessation, there is an improvement in carbohydrate tolerance and insulin function, so that pre-therapy values or even better values are reached [47, 48].

Notably, in our population we found a high incidence of tumours, particularly of cancer, during adulthood. The most frequent form of cancer was papillary thyroid carcinoma, followed by skin cancer and then by CNS cancer. The most frequent form of benign tumour was CNS tumour.

The risk of tumours in women with TS has been little studied and results in the literature are inconclusive, with the exception of the increased risk of gonadoblastoma in the presence of a Y chromosome [49–51], which was confirmed in our study. The few large population-based studies performed on national registries [10–13] and one retrospective study [52] reported that the overall risk of cancer is similar [10–12] or slightly higher in TS [13] with respect to the general population. However, all these studies demonstrate that the pattern of tumour occurrence in TS differs from that of the general population, with an increased risk in TS of benign CNS tumours, particularly meningioma and benign skin neoplasms and skin cancer, particularly melanoma, and a decreased risk of breast cancer. Previous studies have found no increased incidence of thyroid cancer, whereas in our study it was the cancer with the highest incidence. The differences in the frequency of thyroid cancer between our study and others could be explained by the environmental or genetic differences among the populations or, more probably, by the follow-up methods (we performed thyroid ultrasonography at least once every two years). Unlike current practice, this thus suggests the need to include thyroid ultrasonography within the structured monitoring protocol of this syndrome.

In our cohort, in accordance with other studies [12, 52], no association between GH or EPs therapy and tumours was found, suggesting the safety of these replacement therapies in the long term. However, 6 of the 8 cases of papillary carcinoma (75%) were treated with GH, while only 4 of the 8 cases (50%) had HT, thus excluding the potential role of HT in the increased incidence of papillary thyroid carcinoma in these patients [53].

The possible association between GH therapy and papillary carcinoma has been suggested by Cabanas P et al*.* [54], who found GH receptor expression in papillary thyroid carcinoma in two TS children. More data are needed to provide reliable evidence on the possible association between GH therapy and papillary thyroid carcinoma. However, the available data suggest that careful ultrasound thyroid monitoring should be reserved for patients with TS, particularly if treated by GH therapy, regardless of the presence of HT.

Comparative analysis of the karyotype and phenotype revealed an association with hypertension alone. In particular, patients with monosomy 45,X were more likely to have hypertension and those with mosaicism were less likely to have it. Our data are in line with other studies [55]; however, our comparative analysis of the karyotype and phenotype remains inconclusive due to the general uncertainty regarding the extent of mosaicism in different tissues.

In conclusion, this study confirms the need for continued, structured and multidisciplinary monitoring of TS also during adulthood. In addition, it demonstrates that a good transition programme needs to be guaranteed in TS with the participation of all relevant stakeholders to ensure a seamless transfer from paediatric to adult care and adequate treatment and monitoring during adulthood. This could facilitate the early diagnosis of important comorbidities, including cancer, appropriate and timely therapy, the prevention of several health complications, and also help extend the life of these patients. The risk of cancer in women with TS has been little studied, and although the consensus group does not recommend a specific cancer screening protocol, these data suggest the need for the increased surveillance of specific types of cancer in TS, including thyroid cancer. Lastly, the data from this study suggest that a similar management is needed in TS patients regardless of the karyotype, EPs therapy, type of EPs taken (HRT or OCT) and previous use of GH therapy, even if a particular attention to thyroid monitoring is suggested for patients treated by GH.

## Supplementary Information

Below is the link to the electronic supplementary material.

Supplementary file1 (PDF 48 kb)
