# Supplementary material for: New insights into the comorbid conditions of Turner syndrome: results from a long-term monocentric cohort study
Source: J Endocrinol Invest. 2022 Jul 30;45(12):2247–56. doi: 10.1007/s40618-022-01853-z (PMC9646560; doi:10.1007/s40618-022-01853-z)
Supplement: Supplementary file 1 — Supplementary file1 (PDF 48 kb) [file 40618_2022_1853_MOESM1_ESM.pdf]

Supplemental Table 1. Prevalence of comorbid conditions by age group. National data are provided for comparison.

	Study cohort Age group (at follow-up)				National data Age group (Istat 2019)						
	21-34	35-44	45-66	P	20-24	25-34	35-44	45-54	55-59	60-64	65-74
Hypertension	10.6	28.2	41.3	<.001	1.1	1.5	3.6	12	21.4	30	43.3
Type 2 diabetes	6.1	15	41.2	<.001	0.6	0.8	1.4	2.2	5.2	7.1	12.6
Osteoporosis	37.9	20	2.9	<.001	0.6	0.6	1.5	5.5	15.2	21	32.2
N	66	60	34								
